# Quantum Efficiency of Single Dibenzoterrylene Molecules in *p*-Dichlorobenzene at Cryogenic Temperatures

**DOI:** 10.1021/acs.jpcb.3c01755

**Published:** 2023-06-02

**Authors:** Mohammad Musavinezhad, Alexey Shkarin, Dominik Rattenbacher, Jan Renger, Tobias Utikal, Stephan Götzinger, Vahid Sandoghdar

**Affiliations:** † Max Planck Institute for the Science of Light, D-91058 Erlangen, Germany; ‡ Department of Physics, Friedrich Alexander University Erlangen-Nuremberg, D-91058 Erlangen, Germany; § Graduate School in Advanced Optical Technologies (SAOT), Friedrich Alexander University Erlangen-Nuremberg, D-91052 Erlangen, Germany

## Abstract

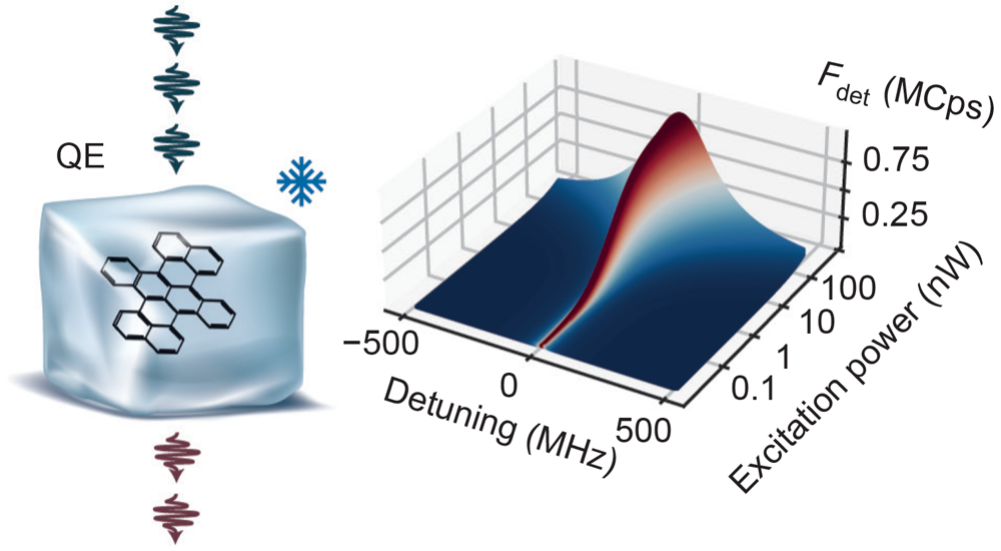

We measure the quantum efficiency (QE) of individual dibenzoterrylene (DBT) molecules embedded in *p*-dichlorobenzene at cryogenic temperatures. To achieve this, we combine two distinct methods based on the maximal photon emission and on the power required to saturate the zero-phonon line to compensate for uncertainties in some key system parameters. We find that the outcomes of the two approaches are in good agreement for reasonable values of the parameters involved, reporting a large fraction of molecules with QE values above 50%, with some exceeding 70%. Furthermore, we observe no correlation between the observed lower bound on the QE and the lifetime of the molecule, suggesting that most of the molecules have a QE exceeding the established lower bound. This confirms the suitability of DBT for quantum optics experiments. In light of previous reports of low QE values at ambient conditions, our results hint at the possibility of a strong temperature dependence of the QE.

## Introduction

Photoluminescence quantum yield (QY) is a key property of an optical emitter, as it determines the emitter’s efficiency of converting the incoming light to luminescence. This quantity plays a crucial role in a variety of applications such as bioimaging^1^ or lasing.^2^ QY is typically defined as the ratio of the number of emitted to absorbed photons and therefore depends on both the emission and absorption properties of the emitter under study.

Given its technological and fundamental importance, there exists an extensive body of work investigating QY in a variety of systems.^3−6^ The great majority of such studies are, however, conducted via ensemble measurements, where a macroscopic quantity of photoluminescent material is typically illuminated with a light of known characteristics. Carefully calibrated measurements of the emission (e.g., using a reference sample of known QY) and absorption (usually determined via thermal effects) are used to extract the QY.^7^ In addition to the challenge of performing accurate calibrations, the very nature of this approach makes it insensitive to interemitter variations that are inherent to the specific emitter type^8,9^ or arise from differences in their local environment, especially in the solid state.^10−12^ As a result, our quantitative and first-principles understanding of the QY remains incomplete.

The recent progress of nano-optics has invoked the use of single quantum emitters in a variety of applications, ranging from biological super-resolution microscopy to quantum information processing.^13^ The photophysics of the emitter and therefore its QY play a central role in nearly all these applications. However, single-molecule QY measurements are very challenging because they require accurate measurements of weak optical powers and minute thermal dissipations. In an alternative approach, one compares the radiated photon rate of a single emitter to the total decay rate of its excited state and defines the quantum efficiency (QE) as
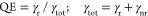
1where γ_r_, γ_nr_, and γ_tot_ denote the radiative, nonradiative, and total decay rates of the given quantum state, respectively. The main difference between QY and QE is that the former is defined for the combination of the emitter and the excitation method, while QE is defined for a given excited state. Hence, while QY is often used as a technologically relevant quantity, QE establishes a fundamental emitter property that can be more readily used in different excitation schemes. In the case of a single-photon excitation with the perfect excitation efficiency (i.e., when all of the decay happens through the chosen state) and single-photon emission both QY and QE provide the same result.

Various methods have been used for measuring QE of single molecules. In one class of experiments,^11^ the collection and detection efficiencies of the measurement setup are carefully calibrated, so that the detected photon rate (power, *P*) can be directly related to the radiative decay rate γ_r_ of the emitter through the relation *P* = ℏωγ_r_ρ_ee_, where ρ_ee_ is the excited state population, ω is the emitted photon frequency, and ℏ is Planck’s constant. In addition, the total decay rate γ_tot_ is directly assessed by measuring the lifetime of the excited state.

Another line of studies is inspired by the pioneering work of Drexhage on the modification of the fluorescence lifetime when an emitter is placed close to an interface.^14^ Here, one exploits the fact that changes to the local electromagnetic environment, e.g., the refractive index of the surrounding,^15^ a movable mirror,^16^ or a tunable optical cavity^10^ modify γ_r_ but leave γ_nr_ unchanged. Because this method only requires lifetime measurements, it circumvents the difficulties associated with the calibration of excitation and emission efficiencies. The downside of the approach is, however, its strong sensitivity to the exact position and orientation of the emitter with respect to the physical boundaries.

In this article, we perform QE measurements on individual dibenzoterrylene (DBT) molecules embedded in an organic crystal (*p*-dichlorobenzene, *p*DCB) at *T* = 2 K.^17^ DBT belongs to the family of polycyclic aromatic hydrocarbons (PAH) which has been used in a number of quantum optical studies because of their high spectral stability, strong zero-phonon lines (ZPL), and negligible dephasing when they are embedded in a suitable matrix at low temperatures.^18^ The results of the experiments have been consistent with high QE values^19−22^ although quantitative QE studies of these systems have been rare.^23^ In fact, explicit reports are missing at the single emitter level. Concrete QE reports of these systems have been based on ensemble^12,24^ or single-molecule^11,16,24^ measurements at room temperature. Interestingly, a recent publication has reported QE values of 35% and below for DBT at room temperature.^24^ The dependence of the fluorescence lifetime and QE on the S_1_–S_0_ transition energy was interpreted in light of the energy gap law (EGL) for the nonradiative decay,^25−27^ which predicts that the internal conversion (IC) rate should grow exponentially as the transition wavelength becomes longer. Because of the near-infrared transition of DBT (between 700 and 800 nm, depending on the matrix),^17,28,29^ EGL is expected to enhance IC and, thus, γ_nr_.^12,26,30^ Our study aims to clarify whether the room-temperature reports for DBT also hold for its cryogenic applications.

## Experimental Methods

The sample preparation is similar to our earlier work.^31^ In short, we first prepare a solution of DBT in *p*DCB at a concentration of about 100 ppm. This solution is then melted (53 °C melting temperature) on a hot plate and introduced into a 1.2 μm thick channel formed between two silica substrates. After crystallization, we perform partial remelting with a slower crystallization step, which produces homogeneous *p*DCB crystals with roughly 100 μm lateral size. Finally, this sample is inserted into the cryostat and cooled below 2 K.

**Figure 1:**
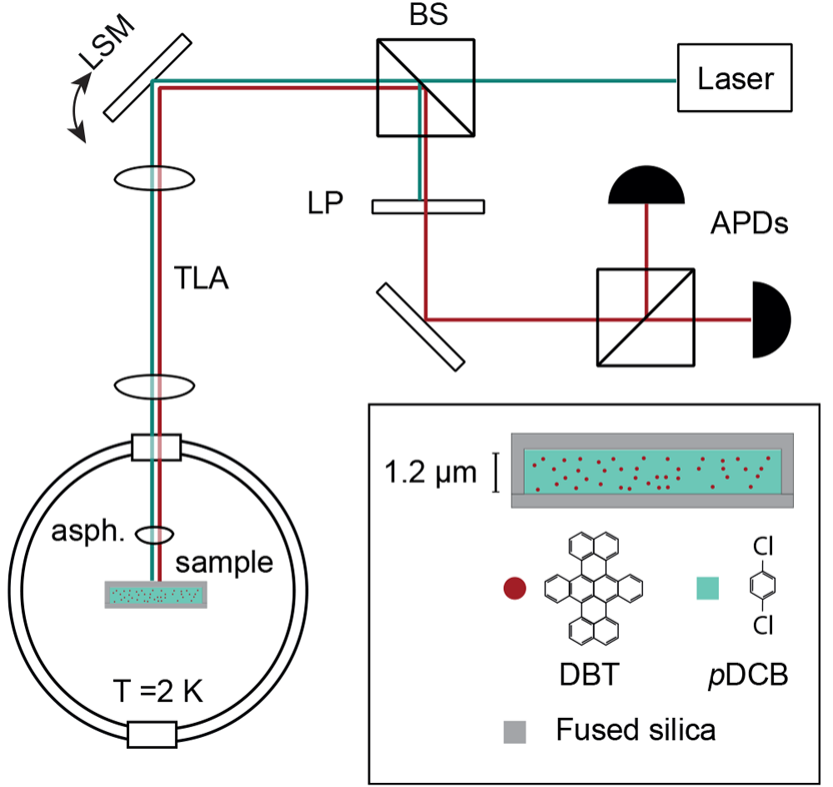
Optical setup and the sample schematics. BS: beam splitter; LSM: laser scanning mirror; TLA: telecentric lens assembly; asph: aspherical lens; LP: long-pass fluorescence filter; APD: avalanche photodiode. The inset shows the cross section of the fused silica channel filled with DBT-doped *p*DCB.

The basis of the experimental setup is a home-built cryogenic confocal microscope, which allows for single-molecule imaging (see Figure 1). To address individual molecules, we excite their narrow lifetime-limited zero phonon lines (typical line width of 25 MHz) using a narrow-band (<1 MHz) continuous-wave Ti:sapphire laser. The laser polarization can be controlled to ensure the best matching to the molecule’s in-plane dipole, and its power can be adjusted using a set of neutral density filters. The coarse sample alignment and the optical focus adjustment are achieved using cryogenic nanopositioners, while the fine positioning of the laser beam can be realized using a laser scanning mirror (LSM) in combination with a telecentric 4*f* lens assembly. The light emitted by the molecule is collected in reflection and sent through a tunable fluorescence filter that blocks the excitation laser but passes the red-shifted fluorescence. This light is then guided onto a pair of avalanche photodiodes (APDs) arranged in a Hanbury Brown and Twiss configuration. This assembly allows us to determine the molecular excited state lifetime and intersystem crossing (ISC) rate via photon autocorrelation measurements (see the Supporting Information).

## Results and Discussion

We measured the properties of 44 molecules at various locations within the sample with transition wavelengths between 743.6 and 745 nm. In order to obtain a fair representation of the overall distribution, we made sure to avoid any selection based on apparent brightness or line width. The only exclusion criterion was the vicinity to cracks in the matrix so as to avoid distorted excitation or emission patterns.

**Figure 2:**
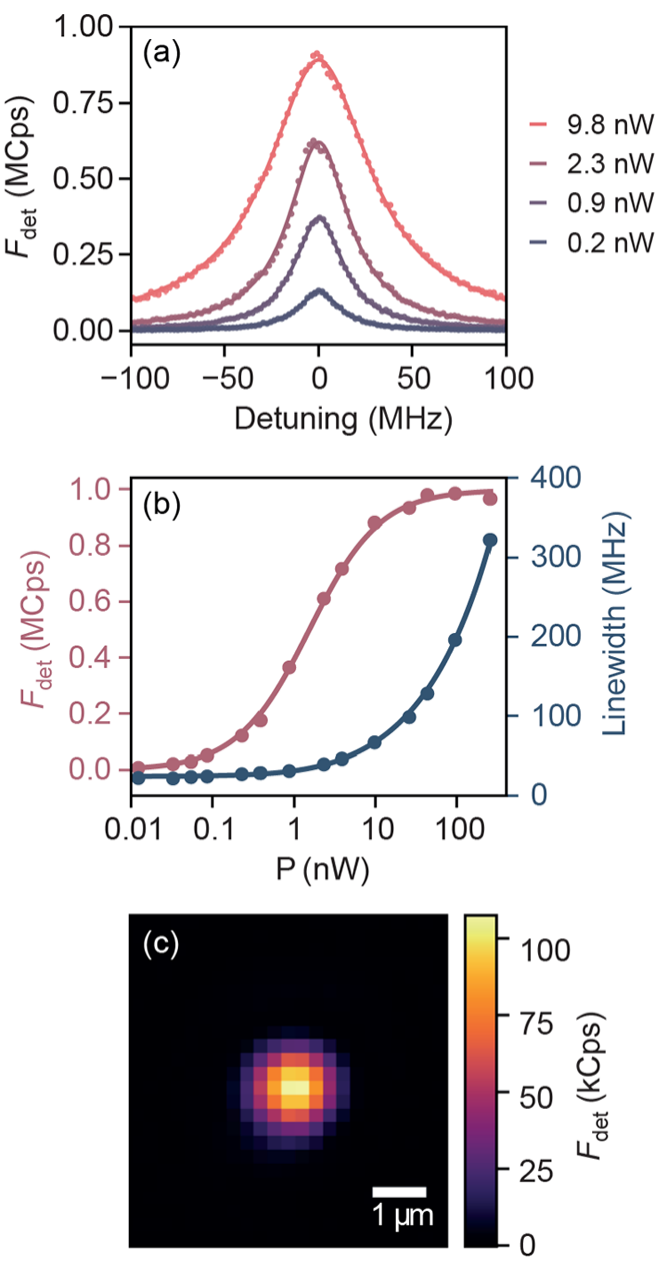
(a) Detected fluorescence counts *F*_det_ of a single molecule as a function of laser ZPL detuning for varying optical power. The molecule ZPL wavelength is λ = 743.7 nm. (b) Fluorescence count rate (red) and line width (blue) plotted against the excitation power. The power is corrected for the setup excitation efficiency η_exc_, and the fluorescence data are corrected for the APD saturation caused by its dead time. The lines are fits to the data according to a semiclassical theory (see the Supporting Information) yielding *F*_det_(∞) = 1.00 MCps and *P*_sat_ = 1.6 nW. (c) Fluorescence excitation point spread function (PSF) of the same molecule. The power was set to *P* = 0.05*P*_sat_ ≪ *P*_sat_ to avoid PSF distortion. The extracted effective illumination area is *A*_eff_ = 2.4 μm^2^.

For each selected molecule, we optimized the incident light polarization and the focus position to achieve the best excitation efficiency and performed a series of laser frequency scans for varying incident powers. As can be seen in Figure 2a, such measurements produce a series of Lorentzians with power-dependent height *F*_det_(*P*) and line width Γ(*P*). This dependence is plotted in Figure 2b and follows the well-known saturation profile, where the line width grows with increasing powers while the emission maximum saturates to a constant value. The results are well described by the standard semiclassical theory for two-level emitters,^32−34^ which provides the expressions (see the Supporting Information)
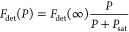
2
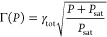
3The formulas depend on the low-power spectroscopic line width γ_tot_, the maximal detected fluorescence count rate *F*_det_(∞), and the saturation power *P*_sat_ at the molecule position. The latter parameters both characterize the strength of the emitter–light interaction and can therefore be used to extract γ_r_. In addition, we can measure γ_tot_ either via the excited state lifetime or directly as the low-power spectroscopic line width of the ZPL under the assumption of negligible dephasing. We, thus, determine the QE of single molecules according to two independent measurement methods.

The first approach is based on analyzing *F*_det_(∞). The semiclassical theory predicts that in our resonant driving scheme at high saturation ρ_ee_ = 1/2 leading to photon emission rate *F*(∞) = γ_r_/2. To relate the detected APD counts *F*_det_ to the fluorescence rate, we analyzed the total detection efficiency of our setup η_tot_ = η_coll_η_tr_η_det_η_spec_. Here, η_coll_ is the collection efficiency of the aspherical lens, η_tr_ accounts for the transmission losses of the various optical elements in the detection path, η_det_ is the APD detection efficiency at 744 nm, and η_spec_ is the effective detected fraction of the emission, including spectral dependence of the detection efficiency and filtering. We extract the above-mentioned efficiencies independently and find η_tr_ = 69% and η_det_ = 55% based on the setup and the APD calibrations. The effective collected spectrum fraction η_spec_ is more difficult to determine because it depends on the chromatic aberrations of the setup (most notably, the aspherical lens), the transmission characteristics of the fluorescence filter, and the drop of the APD detection efficiency with longer wavelengths. Because we need to filter out the excitation laser at the ZPL frequency, the upper limit for η_spec_ in our detection scheme is 1 – α, where α is the emission branching ratio given by the fraction of the emission contained in the ZPL to the total fluorescence, including the red-shifted emission. In our analysis, we keep η_spec_ as an adjustable parameter, which is taken to be the same for all measured molecules. The final parameter, η_coll_, is the least precisely known. Because of the anisotropic dipole emission pattern, η_coll_ is very strongly dependent on the out-of-plane dipole angle θ, varying between η_coll_^H^ = 9% for a horizontally oriented dipole (θ = 0) and η_coll_^V^ = 1% for a vertically oriented dipole (θ = π/2; see the Supporting Information). In general, η_coll_ can be expressed as a weighted combination η_coll_(θ) = η_coll_^H^ cos^2^(θ) + η_coll_^V^ sin^2^(θ). Unfortunately, the preparation method of our sample does not produce predefined molecule orientations.^35,36^ Moreover, the relatively low numerical aperture of our collection lens (0.77) does not let us estimate θ.^37^ As a result, the measured QE values can vary by almost an order of magnitude depending on the orientation of a molecule. Hence, this measurement scheme only provides a lower bound for the QE.

The second QE measurement method relies on the saturation power *P*_sat_. As we show in the Supporting Information, *P*_sat_ can be related to the radiative decay rate γ_zpl_ = αγ_r_ that takes place via ZPL as
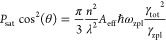
4where *n* is the refractive index of the host matrix, λ is the vacuum wavelength of the ZPL, ω_zpl_ is the corresponding angular transition frequency, and *A*_eff_ is the effective area of the excitation beam. Given this equation and the knowledge of the branching ratio α, we can calculate γ_zpl_ from *P*_sat_ and then relate it to the total radiative decay rate γ_r_. As in the previous method, we can extract γ_tot_ from the spectroscopic line width at low excitation powers. The transition frequency is readily obtained from high-resolution fluorescence scans. The parameter *n* is more difficult to estimate due to the birefringence of the host material and the uncertainty in the reference data;^38^ in our calculations we have assumed *n* = 1.6.

To measure the effective mode area *A*_eff_, we used the molecule itself as a local intensity probe. Here, we employed the LSM to raster scan the position of the resonant focused beam over the molecule while recording its fluorescence. As long as the laser power is significantly below saturation, the fluorescence is proportional to the local optical intensity, and the measured data directly provide the intensity distribution *I*(**r**). An example of a resulting map is shown in Figure 2c. Next, we evaluate *A*_eff_ = ∫*I*(**r**)d*A*/*I*(**r**_mol_), where **r**_mol_ is the molecule’s position. To ensure that the focused light is transversely polarized and that there were no clipping losses at the aspherical lens, we reduced the diameter of the incident laser beam. We note that the main source of uncertainty in this method is again the angle θ between the molecular dipole and the substrate plane. However, because θ affects the QE measurements following the same trend in both methods, it still lets us put an estimate on other sources of uncertainty, which are mostly different between the two methods (see the Supporting Information).

Before we present the experimental data, we mention that we have neglected several effects in the analysis described above. First, we do not include the contribution of the ISC,^39,40^ which involves long-lived shelving states and is power dependent. However, our intensity autocorrelation measurements confirm that the effect of ISC on the emission rate is as low as 0.1%, consistent with the literature knowledge that the ISC yield is extremely low for DBT in *p*DCB at cryogenic temperatures^30^ (see the Supporting Information). Second, we did not take into account any dephasing, as we reached the line width reported for DBT in *p*DCB at *T* < 2 K, consistent with the measured lifetimes of the excited state.^17,41^ We have confirmed this by extracting the excited state lifetime from the same intensity autocorrelation measurements (see the Supporting Information).

**Figure 3:**
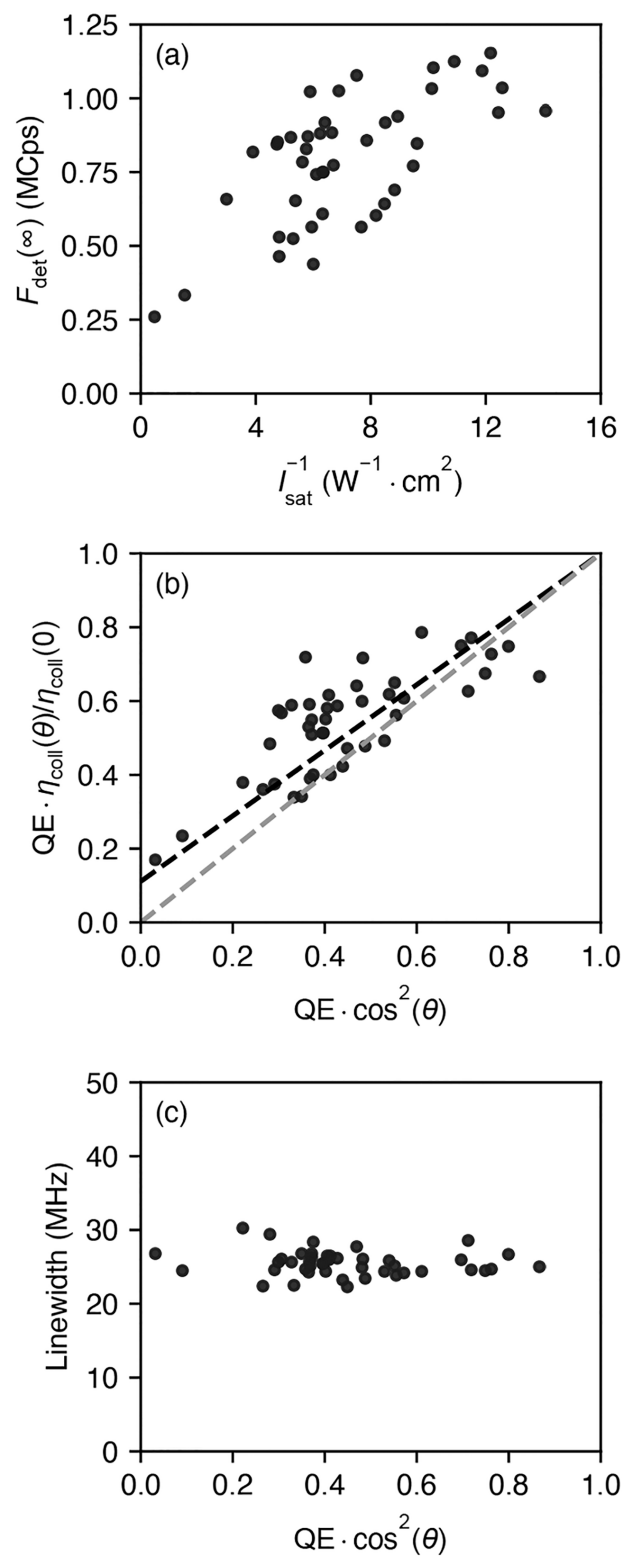
(a) Joint distribution of the saturation fluorescence count rates and the inverse saturation power for the analyzed molecules. (b) Same, but for the extracted “effective” QE, which includes dipole orientation factors. The black and the gray dashed lines show the expected values assuming perfect QE = 1 with varying 0 ≤ θ ≤ π/2 or perfect orientation θ = 0 with 0 ≤ QE ≤ 1, respectively. (c) Joint distribution of the spectroscopic line width and the effective QE.

Figure 3 summarizes the results obtained for 44 individual molecules. In Figure 3a, we show the recorded fluorescence rate as a function of the inverse saturation intensity *I*_sat_^–1^ = *A*_eff_/*P*_sat_. The values are well correlated, which is to be expected as both are equal to the product of the QE and a geometric θ-dependent factor. Moreover, the results span over a large range of emission rates and saturation powers, indicating strong heterogeneity of the measured molecules. Although the QE of the individual molecules might differ, the observed strong heterogeneity likely stems from the variations in dipole orientation.

It is instructive to examine the observations at the two ends of the range in Figure 3a. On the left side, there is a molecule with very high saturation intensity (more than 13 times above the median), which still produces relatively high saturation counts of 0.25 MCps. This is consistent with an almost perfectly out-of-plane dipole, which is hard to excite with transverse-polarized light, but whose fluorescence can still be collected with a finite efficiency η_coll_ ≈ 1%. On the right side, there are molecules with very high fluorescence count rates >1.1 MCps and correspondingly low saturation intensities, suggesting that a significant fraction of molecules has a high QE.

Next, we use individually measured values of γ_tot_ and the knowledge of the setup calibration to examine the QE. As mentioned earlier, both methods rely on the dipole orientation. Therefore, in Figure 3b, we represent the results of the first and second methods via QE η_coll_(θ)/η_coll_(0) and QE cos^2^(θ), respectively. The two quantities are equal to the QE for the most favorable horizontal dipole (θ = 0) and underestimate the QE for other orientations. The best agreement between the two methods is obtained if we take α = 0.33 and assume that η_spec_ = 0.8(1 – α), i.e., if the detection efficiency for the non-ZPL fluorescence is 80% of that for ZPL contribution. This is reasonable considering the chromatic response of the collection and detection system over a spectral range of 100 nm; based on our previous estimates of the DBT emission spectra,^41^ measurements of the fluorescence filter transmission, and APD calibrations, we expect at least 40% collection efficiency of the red-shifted fluorescence compared to the resonant light, i.e., η_spec_ > 0.4(1 – α). A branching ratio of 33% is also consistent with previously reported experimental values^17^ and theoretical estimates^42^ although a relative error of up to 50% might be at play.

To further illustrate the connection between the two quantities plotted in Figure 3b, we also depict their expected relationship in the two limiting cases. In the first limit depicted by the lower-lying gray dashed line, we assume the perfect dipole orientation θ = 0, in which case the two quantities are simply equal to QE. In the second limit denoted by the higher-lying black dashed line, the QE is taken to be 1, so that the observed intermolecule variation is purely due to different values of θ. In this case, the horizontal axis denotes cos^2^(θ), while the vertical axis presents η_coll_(θ)/η_coll_(0) = η_coll_^V^/η_coll_^H^ + (1 – η_coll_^V^/η_coll_^H^) cos^2^(θ). One can see that there is still a linear relationship between the two quantities, with the nonzero vertical axis intercept determined by the ratio of the collection efficiencies for the two dipole orientations η_coll_^V^/η_coll_^H^, which is about 0.1 for our system. This reflects the fact that the perfect out-of-plane dipole (θ = π/2) cannot be excited in our configuration but can still be detected because of a finite value of η_coll_^V^. In the absence of any experimental uncertainty, we expect the data to lie between the two dashed lines.

The data points in Figure 3b are spread over a wide range, while a nonnegligible fraction lies above 70%. We estimate the overall uncertainty in our measurements to be about 20% (see the Supporting Information), which is substantially smaller than the variations observed in Figure 3. Hence, we conclude that the QE of DBT in *p*DCB may assume large values in the range of 70 ± 20%. In fact, in one single case (not shown in Figure 3b), the extracted QE value significantly exceeded 1 for both methods used in this article. We suspect that the orientation and position of that molecule with respect to its surrounding matrix and substrate happened to inflict a planar antenna effect on it, leading to an increase in the excitation and collection efficiency.^11^ We have excluded that molecule from our analysis.

Figure3c shows that the measured distribution of the line widths γ_tot_ is much narrower than the distribution of the data points presented in Figure 3b and that there is no clear correlation between the two. Because we do not expect substantial variations of γ_r_ among the different molecules in the crystal, this observation can only be reconciled with the relation γ_tot_ = γ_r_/QE via the distribution of the dipole orientation θ.

## Conclusions

We have analyzed the quantum efficiency of DBT in *p*DCB at liquid helium temperature using two different methods. The results agree well within reasonable assumptions about the branching ratio and the fluorescence collection efficiency, but in both cases we find a large spread in the extracted QE values. We attribute this distribution to the variation in the orientations of the individual molecules. Our analysis suggests that the QE of DBT reaches above 70%. The discrepancy with the recently reported low QE values of DBT at ambient conditions^24^ can be attributed to a number of effects. First, QE might experience a significant temperature dependence. Second, as has been discussed in the literature of the local field effects, the radiative decay rate is a nontrivial function of the refractive index and the molecular nature of the way an emitter is embedded in its environment.^43−45^ Similarly, the nonradiative decay rate is known to depend on the chemical make of the surrounding medium. Future studies should pursue comprehensive measurements using the very same system under different conditions. Furthermore, quantification of the dipole orientation will be invaluable for more precise measurements, e.g., by employing high-NA optics or planar optical antennas^11^ for collecting all polarizations. Finally, uncertainties in the branching ratio and host matrix birefringence and refractive index should be characterized more accurately.

## Supporting Information Available

Derivation of the two QE extraction methods including eq 4, detailed description of the setup calibration, discussion of the effects of α and η_spec_ on the final result, intensity autocorrelation measurements, and analysis of the uncertainty sources (PDF)
