# Supplementary material for: Quantum Efficiency of Single Dibenzoterrylene Molecules in p-Dichlorobenzene at Cryogenic Temperatures
Source: J Phys Chem B. 2023 Jun 2;127(23):5353–9. doi: 10.1021/acs.jpcb.3c01755 (PMC10278129; doi:10.1021/acs.jpcb.3c01755)
Supplement: Supplementary file 1 — jp3c01755_si_001.pdf [file jp3c01755_si_001.pdf]

Quantum Efficiency of Single Dibenzoterrylene Molecules in para-Dichlorobenzene at Cryogenic Temperatures

Mohammad Musavinezhad,[1, 2] Alexey Shkarin,[1] Dominik Rattenbacher,[1] Jan Renger,[1] Tobias Utikal,[1] Stephan Götzinger,[2, 1, 3] and Vahid Sandoghdar[1, 2, *]

[1] *Max Planck Institute for the Science of Light, D-91058 Erlangen, Germany*
[2] *Department of Physics, Friedrich Alexander University Erlangen-Nuremberg, D-91058 Erlangen, Germany*
[3] *Graduate School in Advanced Optical Technologies (SAOT), Friedrich Alexander University Erlangen-Nuremberg, D-91052 Erlangen, Germany*

I. MODEL FOR MOLECULE EXCITATION AND SATURATION

In the semi-classical theory of light-matter interaction, the Hamiltonian in the rotation wave approximation and in the frame rotating with the driving frequency ω is given by

$$\hat{H} = -\frac{\hbar\Delta}{2}\hat{\sigma}_z + \frac{\hbar\Omega}{2}\left(\hat{\sigma}_+ + \hat{\sigma}_-\right), \tag{1}$$

where $\Delta = \omega - \omega_0$ is the difference between the frequency ω of the applied electric field $\boldsymbol{E}$ and the molecule's transition frequency ω_0, $\Omega = -\frac{\boldsymbol{d}\cdot\boldsymbol{E}}{\hbar}$ is the Rabi frequency, and $\boldsymbol{d}$ the transition dipole moment of the molecule. The operators are defined as $\hat{\sigma}_+ = |e\rangle\langle g|$, $\hat{\sigma}_- = |g\rangle\langle e|$ and $\hat{\sigma}_z = |e\rangle\langle e| - |g\rangle\langle g|$. The master equation for the density matrix $\hat{\rho}$ is then given by

$$\frac{\mathrm{d}\hat{\rho}}{\mathrm{d}t} = \frac{1}{\mathrm{i}\hbar}\left[\hat{H},\hat{\rho}\right] + \mathcal{L}[\hat{\rho}] \quad \text{with the Liouvillian}$$
$$\mathcal{L}[\hat{\rho}] = \frac{\gamma_{\mathrm{tot}}}{2}\left(2\hat{\sigma}_-\hat{\rho}\hat{\sigma}_+ - \hat{\sigma}_+\hat{\sigma}_-\hat{\rho} - \hat{\rho}\hat{\sigma}_+\hat{\sigma}_-\right), \tag{2}$$

which describes the spontaneous decay of the excited state due to the coupling to a Markovian reservoir of optical modes. The total decay rate of the excited state γ_{tot} is influenced by three main decay channels. The first is the decay into the 0-0 zero-phonon line (ZPL) associated with the emission of a coherent photon. In the second case, the excited state relaxes into the vibrational manifold of the electronic ground state via emission of a redshifted photon. And lastly, all nonradiative relaxations of the excited state contribute to the total decay rate and reduce the quantum efficiency QE $= \eta$. Therefore, we can write $\gamma_{\mathrm{tot}} = \gamma_{\mathrm{ZPL}} + \gamma_{\mathrm{fluor}} + \gamma_{\mathrm{nr}}$, with

$$\gamma_{\mathrm{ZPL}} = \frac{\omega_0^3|\boldsymbol{d}|^2}{3\pi\epsilon_0\hbar c^3}nf^2(n), \tag{3}$$

where $f(n)$ is the local field correction for an electric dipole transition in a dielectric medium with refractive index n [1–4]. Depending on the polarizability of the emitter compared to the matrix, this correction can be taken as either $f(n) = 3n^2/(2n^2+1)$ ("empty cavity" correction) or $f(n) = (n^2+2)/3$ ("virtual cavity" correction) [5], which can be different by about 50 % for our parameters. As we will discuss later, this correction cancels out in the central expression of concern in this work. Therefore, we leave it as a generic expression and do not elaborate on its exact form.

The photon emission rate of a single molecule is given by $F = (\gamma_{\mathrm{ZPL}} + \gamma_{\mathrm{fluor}})\rho_{ee}^{ss} = \eta\gamma_{\mathrm{tot}}\rho_{ee}^{ss}$, where ρ_{ee}^{ss} is the steady state population of the excited state. Defining the saturation parameter as $S = P/P_{\mathrm{sat}} = 2\Omega^2/\gamma_{\mathrm{tot}}^2$, the excited state population can be written as

$$\rho_{ee}^{ss} = \frac{1}{2}\frac{S}{1 + S + \left(2\Delta/\gamma_{\mathrm{tot}}\right)^2}. \tag{4}$$

* vahid.sandoghdar@mpl.mpg.de

This derivation assumes negligible dephasing, which is justified at cryogenic temperatures for DBT molecules embedded in pDCB.

Generally, the measured linewidth Γ can be larger than the pure radiative linewidth at weak excitation $\gamma_r = \eta\gamma_{\mathrm{tot}}$ due to a finite nonradiative decay rate. Additionally, at high excitation powers, the emission rate saturates and the measured linewidth increases via

$$F(S) = F(\infty)\frac{P}{P + P_{\mathrm{sat}}} \quad \text{and} \quad \Gamma(S) = \gamma_{\mathrm{tot}}\sqrt{\frac{P + P_{\mathrm{sat}}}{P_{\mathrm{sat}}}}, \tag{5}$$

respectively. For $S = 1$, i.e. $P = P_{\mathrm{sat}}$, the relation between the molecule decay rate and the electric field at the emitter position is given by

$$\gamma_{\mathrm{tot}}^2 = 2\Omega^2 = 2\frac{\cos^2(\theta)|d|^2|\boldsymbol{E}_{\mathrm{sat}}|^2}{\hbar^2}, \tag{6}$$

where θ is the angle between the transition dipole orientation and the electric field polarization. Using Eq. (3) and Eq. (6), the electric field amplitude at saturation $S = 1$ can be written as

$$|\boldsymbol{E}_{\mathrm{sat}}|^2 = \frac{\hbar}{2}\frac{\omega_0^3}{3\pi\epsilon_0 c^3}\frac{1}{\cos^2(\theta)}\frac{\gamma_{\mathrm{tot}}^2}{\gamma_{\mathrm{ZPL}}} n f^2(n). \tag{7}$$

In the next step, we relate the electric field amplitude at the position of the molecule to directly measurable parameters. Assume a total illumination power P, focused onto the vicinity of the molecule positioned at $\boldsymbol{r} = \boldsymbol{r}_{\mathrm{m}}$. For excitation intensities significantly lower than saturation, i.e. $S \ll 1$, the fluorescence count rate is linearly proportional to the electric field intensity at the position of the molecule, such that $F_{\mathrm{det}} \propto |\boldsymbol{E}(\boldsymbol{r}_{\mathrm{m}})|^2 \propto I(\boldsymbol{r}_{\mathrm{m}})$. We use a laser scanning mirror (LSM), to map the intensity distribution of the excitation beam, and define the effective beam area A_{eff} such that $A_{\mathrm{eff}} = \int I(\mathbf{r})dA/\mathrm{I}(\mathbf{r}_{\mathrm{m}}) = P/I(\mathbf{r}_{\mathrm{m}})$. As a result, A_{eff} can be directly measured through

$$A_{\mathrm{eff}} = \frac{\int F_{\mathrm{det}}(\boldsymbol{r})d^2\boldsymbol{r}}{F_{\mathrm{det}}(\boldsymbol{r}_{\mathrm{m}})}, \tag{8}$$

where $F_{\mathrm{det}}(\boldsymbol{r})$ is the detected fluorescence count rate, when the beam center is at position $\boldsymbol{r}$ in the sample plane.

The excitation intensity $I(\mathbf{r}_{\mathrm{m}})$ is linked to the macroscopic electric field in the medium via

$$I(\mathbf{r}_{\mathrm{m}}) = \frac{cn\epsilon_0}{2}|\boldsymbol{E}_{\mathrm{macro}}|^2, \tag{9}$$

Thus,

$$|\boldsymbol{E}_{\mathrm{macro}}|^2 = \frac{2}{cn\epsilon_0}\frac{P}{A_{\mathrm{eff}}}. \tag{10}$$

The local field strength at the molecule position $|\boldsymbol{E}(\boldsymbol{r}_m)|^2$ is obtained by multiplication with the local field correction factor [3]

$$|\boldsymbol{E}(\boldsymbol{r}_m)|^2 = |\boldsymbol{E}_{\mathrm{macro}}|^2 f^2(n). \tag{11}$$

Substituting the electric field intensity in Eq. (7) with the measurable parameters given by Eq. 11, yields

$$\begin{aligned} |\boldsymbol{E}_{\mathrm{sat}}|^2 &= \frac{\hbar}{2}\frac{\omega_0^3}{3\pi\epsilon_0 c^3}\frac{1}{\cos^2(\theta)}\frac{\gamma_{\mathrm{tot}}^2}{\gamma_{\mathrm{ZPL}}} n f^2(n) \\ &= \frac{2}{cn\epsilon_0}\frac{P_{\mathrm{sat}}}{A_{\mathrm{eff}}} f^2(n), \end{aligned}$$

which can be rewritten as

$$P_{\rm sat}\cos^2(\theta) = \frac{\pi}{3}\frac{n^2}{\lambda^2}A_{\rm eff}\hbar\omega_0\frac{\gamma_{\rm tot}^2}{\gamma_{\rm ZPL}}. \tag{12}$$

It is important to note that this final expression is independent of the local field correction factor $f(n)$, even though both the local applied field in Eq. (11) and the radiative decay rate in Eq. (3) include it. This is to be expected, as this correction equally affects the real driving field in the saturation power calculation and the vacuum fluctuations of the electric field responsible for the spontaneous decay.

II. ESTIMATION OF THE MAXIMUM EMISSION RATE

For $S \gg 1$, the steady state population of the excited state saturates and from Eq. (4) and Eq. (5) we get

$$F(\infty) = \lim_{S\to\infty} F(S) = \lim_{S\to\infty} \gamma_r \rho_{ee}^{ss} = \frac{1}{2}\gamma_r. \tag{13}$$

We account for a non-ideal detection efficiency by introducing $\eta_{\rm tot}$ the total detection efficiency. This allows us to relate the detected count rates $F_{\rm det}(\infty)$ to the total emission rate by

$$\begin{aligned} F_{\rm det}(\infty) =& \eta_{\rm tot}F(\infty) \\ =& \eta_{\rm coll}\eta_{\rm tr}\eta_{\rm spec}\eta_{\rm det}F(\infty), \end{aligned} \tag{14}$$

where we introduce $\eta_{\rm coll}$ for the collection efficiency of the aspherical lens, $\eta_{\rm tr}$ for the transmission losses in the detection path, $\eta_{\rm spec}$ is the effective fraction of the emission spectrum detected by the APD, and $\eta_{\rm det}$ is the APD detection efficiency.

The transmission efficiency of our setup is measured to be $\eta_{\rm tr} = 69\%$. To quantify the APD detection efficiency, a calibrated ND filter is used to attenuate known laser powers. Comparing the expected count rates with measured values by the APD, the quantum efficiency of the APD is found to be $\eta_{\rm det} = 55\%$ at 744.5 nm. APD's quantum efficiency further decreases with longer wavelengths, which is taken into account in $\eta_{\rm spec}$.

To calculate the collection efficiency of the aspherical lens $\eta_{\rm coll}$, we decompose a dipolar radiation pattern into plane waves propagating in the sample (Fig. S1). In order to calculate the fraction of radiation intensity that falls within the solid angle collected by the aspherical lens, the orientation of the emitter must be considered. Accordingly, assuming an emitter located in the center of the pDCB layer with an out of plane angle θ, we estimate the collection efficiency as $\eta_{\rm coll} = \eta_{\rm coll}^H \cos^2(\theta) + \eta_{\rm coll}^V \sin^2(\theta)$ with $\eta_{\rm coll}^H = 9\%$ and $\eta_{\rm coll}^V = 1\%$. We also note that changing the molecule's distance from the glass-pDCB interface did not affect these values by more than 10%. A more detailed discussion of this method is given in Ref. [6].

Finally, we account for the effective spectral detection window $\eta_{\rm spec}$. We consider three main sources that prevent $\eta_{\rm spec}$ from reaching 100%. First, the long pass filter (LP) rejects all coherent emissions, limiting $\eta_{\rm spec}$ to a maximum of $(1-\alpha)$, where α is the fraction of photons emitted coherently through ZPL. Second, due to the finite slope of the tunable LP, photons spectrally close to the ZPL cannot be detected because the filter edge is set such that the excitation beam is efficiently blocked (see Fig. S2). Third, due to the aspheric lens' chromatic aberrations, the redshift photons are not perfectly collimated, which results in extra clipping losses at longer wavelengths. Additionally, as the wavelength increases, the APD's quantum efficiency decreases further. In the following section, we will discuss how to estimate $\eta_{\rm spec}$.

III. SPECTRAL DETECTION WINDOW AND THE BRANCHING RATIO

Determining the maximum emission rate $F(\infty)$ requires knowledge of the effective spectral detection window $\eta_{\rm spec}$. In order to estimate this value, we separate the chromatic dependencies from the

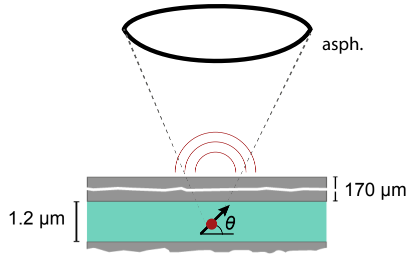

FIG. S1. Calculation of the collection efficiency η_{coll}. The emitter is assumed to be at the center of the pDCB layer (green) with $n = 1.6$, within a channel made in fused silica with $n = 1.45$. asph: Aspherical lens, NA$= 0.77$.

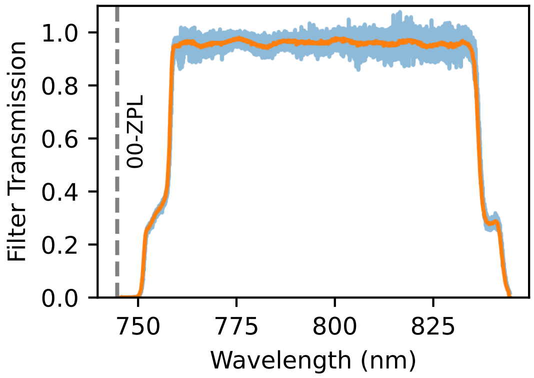

FIG. S2. Transmission profile of the long pass fluorescence filter. Blue line shows the normalized photon counts. Orange line is the rolling average of the raw data. Filter edge is adjusted to reject the excitation beam (dashed line).

branching ratio by defining $\eta_{\text{spec}} = \hat{\eta}_{\text{spec}}(1-\alpha)$. To independently demonstrate the effects of these two parameters, we have performed exactly the same analysis as was done for Fig. 3(b) in the main text, but this time varying the values of $\hat{\eta}_{\text{spec}}$ and α. The results are demonstrated in Fig. S3, and the exact analysis procedure is described below.

The values on the x axis are calculated by substituting $\text{QE} = \gamma_r/\gamma_{\text{tot}} = \gamma_{\text{ZPL}}/(\alpha\gamma_{\text{tot}})$ in Eq. (12) such that

$$\text{QE} \cdot \cos^2(\theta) = \frac{1}{\alpha}\frac{\pi}{3}\frac{n^2}{\lambda^2}\frac{A_{\text{eff}}}{P_{\text{sat}}}\hbar\omega_0\gamma_{\text{tot}}, \tag{15}$$

where γ_{tot} and ω_0 are directly measured as the low-power spectroscopic linewidth and resonance frequency respectively (see Fig. 2(b)). The saturation power P_{sat} is derived from fitting Eq. (5) to the saturation measurement similar to the ones in Fig. 2(b), and A_{eff} is extracted from the LSM measurements (see Eq. (8) and Fig. (2)c). The unknown value of θ in this expression is the angle between emitter's transition dipole vector and polarization of the excitation beam. In our measurements, we have adjusted the in-plane angle of the incident polarization to maximize the excitation of the emitter. As a result, the angle θ in Eq. (15) is the out of plane angle of the emitter.

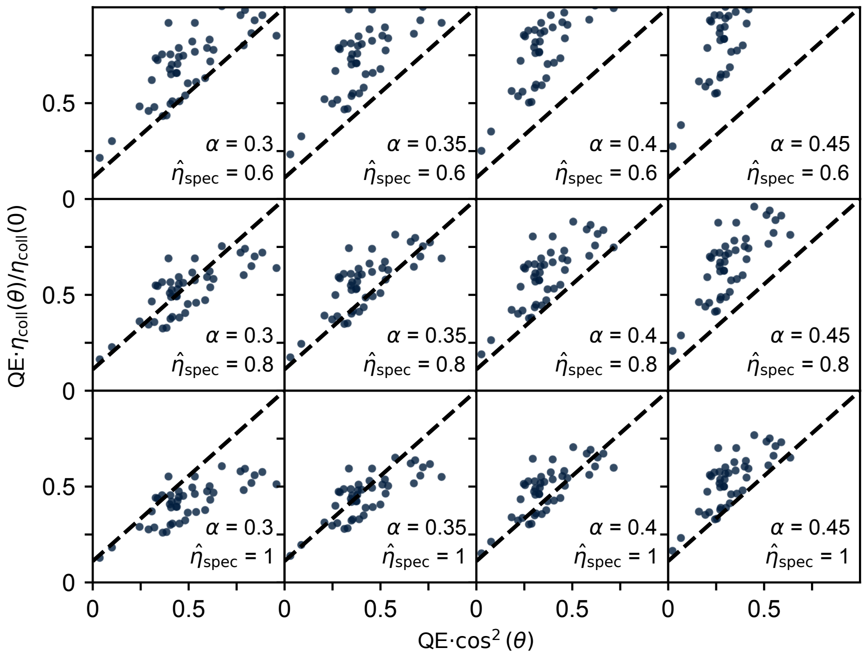

FIG. S3. Estimation of the effective spectral detection window and the branching ratio. Comparing the predicted QE of individual molecules, the best agreement between the two methods occurs for $\hat{\eta}_{\rm spec} \approx 0.8$ and $\alpha \approx 0.33$.

The y axis values in Fig. S3 are calculated using Eq. (13) and Eq. (14) via

$$\begin{aligned} \mathrm{QE} \cdot \frac{\eta_{\rm coll}(\theta)}{\eta_{\rm coll}(0)} &= \frac{\gamma_r}{\gamma_{\rm tot}} \frac{\eta_{\rm coll}(\theta)}{\eta_{\rm coll}(0)} \\ &= \frac{2}{\gamma_{\rm tot}} \frac{F_{\rm det}(\infty)}{\eta_{\rm coll}(\theta)\eta_{\rm tr}\eta_{\rm spec}\eta_{\rm det}} \frac{\eta_{\rm coll}(\theta)}{\eta_{\rm coll}(0)} \\ &= \frac{2}{\gamma_{\rm tot}} \frac{F_{\rm det}(\infty)}{\eta_{\rm tr}\hat{\eta}_{\rm spec}(1-\alpha)\eta_{\rm det}\eta_{\rm coll}(0)} \end{aligned} \tag{16}$$

with $F_{\rm det}(\infty)$ derived from the saturation measurement. The other parameters on the right side of the equation are also known as explained above. Here, θ denotes emitter's out of plane angle (Fig. S1), which is the same angle as in Eq. (15).

As illustrated in Fig. S3 and described in Eq. (15) and Eq. (16), by increasing the branching ratio, the predicted QE via saturation measurement (x axis) decreases, whereas the corresponding values derived from fluorescence counts (y axis) increase. Changes in the chromatic factor $\hat{\eta}_{\rm spec}$, however, only shift the y axis.

Considering that $\hat{\eta}_{\rm spec} < 1$ (since the phonon wing is filtered and the APD's quantum efficiency drops by more than 10% at longer wavelength), the best agreement between the two methods occurs for $\hat{\eta}_{\rm spec} \approx 0.8$ and $0.30 < \alpha < 0.35$. This leads us to estimate $\eta_{\rm spec} \approx 0.8(1-0.33)$.

IV. INTENSITY AUTOCORRELATION MEASUREMENTS

In order to justify our assumptions about negligible effects of dephasing and inter-system crossing (ISC), we have performed detailed measurements of the intensity (i.e., second-order) autocorrelation function for a single randomly selected molecule. These measurement were done for a range of resonant (i.e., via ZPL) excitation powers. The results are displayed in Fig. S4.

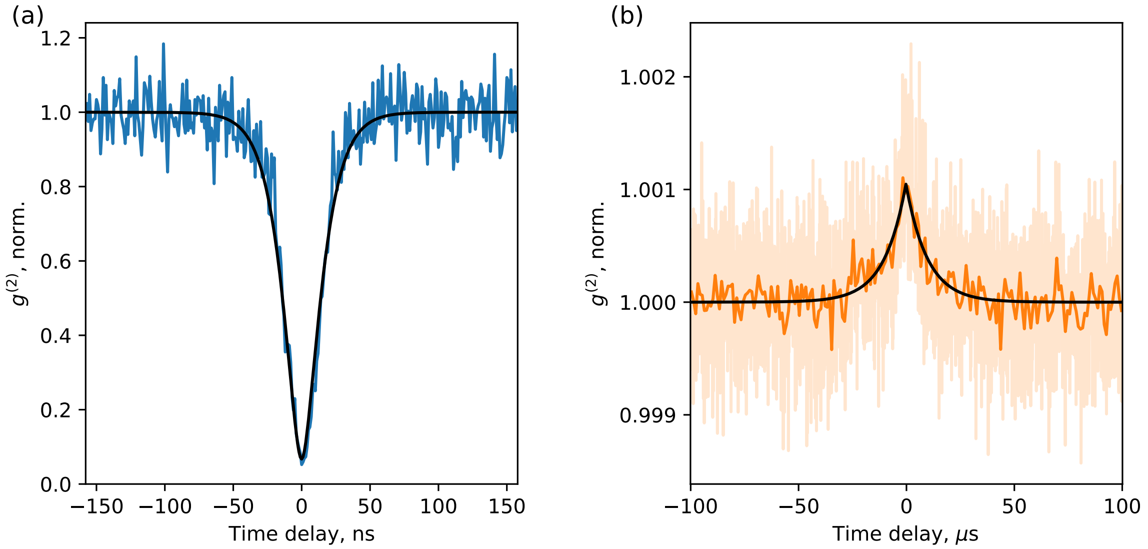

FIG. S4. (a) Intensity autocorrelation function $g^{(2)}(\tau)$ for a molecule fluorescence at short timescales. The molecule was excited via its ZPL at low power $P \approx 0.05P_{\mathrm{sat}}$. The black line is a theory fit as discussed in the text. (b) Same, but for longer timescales and for higher power $P \approx 200P_{\mathrm{sat}}$. The darker orange line depicts the data binned in $1\,\mu$s-wide bins, while the light-orange line corresponds to the bin size of $0.1\,\mu$s. The black line is an exponential fit to the light-orange data as described in the text. The data in the interval $[-0.1\,\mu\mathrm{s}, +0.1\,\mu\mathrm{s}]$ has been removed for plotting and fitting.

The first plot (Fig. S4(a)) shows the autocorrelation function obtained for short time scales on the order of the excited state lifetime, and at a very low excitation power $P \approx 0.05P_{\mathrm{sat}} \ll P_{\mathrm{sat}}$. The data is fitted by a theoretical expression which takes into account saturation and dephasing effects[7]. In the fit we have fixed the values of the low-power homogeneous linewidth to 25.2 MHz and the power-broadened linewidth to 25.5 MHz, which have been measured separately. The best fit, as shown by the black line, was obtained by setting the lifetime-limited linewidth to be equal to the homogeneous linewidth, which confirms negligible dephasing.

The second plot (Fig. S4(b)) conveys the same kind of information, but for longer timescales and for an excitation power well above saturation, $P \approx 200P_{\mathrm{sat}} \gg P_{\mathrm{sat}}$. The plot shows a bunching peak, which can be well fitted by a simple exponential decay function $g^{(2)}(\tau) = 1 + Ce^{-|\tau|/\tau_0}$. Such a peak is indeed characteristic of a long-lived shelving triplet state. However, its very low contrast of $C = 10^{-3}$ indicates that the fraction of time spent in the shelving state $C/(1+C) \approx C = 10^{-3}$ is negligible, which means that the ISC effects can be ignored in our QE analysis. Furthermore, in this regime the exponent decay time of $\tau_0 = 10\,\mu\mathrm{s}$ is almost equal to the triplet state lifetime. Together with the contrast, this lets us estimate the shelving rate of $C/\tau_0 \approx 2\pi \cdot 15$ Hz corresponding to a triplet state yield of 10^{-6}. Both the triplet state lifetime and its yield are in a relatively good agreement with the values previously obtained for DBT in anthracene at low temperatures[8].

V. SOURCES OF UNCERTAINTY

In this section we list and estimate potential sources of uncertainty for both methods. We omit the sources already discussed in the main text and in the previous section, i.e., the dipole orientation, the ISC, and any extra dephasing.

First, we discuss the fluorescence detection method, which mostly relies on the knowledge of the collection efficiency and the detection losses. The calculated values depend on the following factors:

- The calibration uncertainties. We have estimated the uncertainty in the setup transmission

efficiency η_{tr} at 744.5 nm to be around 0.2%, and the uncertainty in APD detection efficiency η_{det} at this wavelength at around 2%. As a result, the total absolute uncertainty due to these sources is about 4 %.

- The dipole emission collection efficiency. It depends on the dielectric environment of the molecule, including the nanochannel thickness, the molecule's position within the nanochannel, and the refractive index of the host crystal and the nanochannel material. None of these values (save for the nanochannel refractive index) can be precisely determined due to local fabrication variations, random molecule distribution, and uncertainty in the host crystal orientation and material parameters. Nevertheless, calculations and numerical simulations for different combinations of these parameters do not produce more than ±10 % deviation. Furthermore, most of this deviation varies from molecule to molecule, showing up as a spread in their QEs rather than a constant systematic deviation.

- The extra wavelength-dependent fluorescence losses. They depend both on the exact chromatic distortions of the optical setup and on the spectral power distribution of the fluorescence, neither of which are known precisely. In practice, we leave it as a fit parameter in our analysis and get a value of $\hat{\eta}_{\mathrm{spec}} = 0.8$. This is a reasonable number given the relatively large wavelength span of the fluorescence emission.

Conversely, the excitation method relies on the precise determination of the incident laser intensity, but is insensitive to the detection efficiency. The local intensity is affected by the following factors:

- Reflection and interference of the excitation light. In calculating the intensity experienced by the molecule, we have assumed that the focused light passes the molecule only once, i.e., there are no reflections at interfaces. However, these reflections do happen in practice, and the resulting back-scattered light interferes with the incident light producing a standing wave. This means that molecules can experience a different optical intensity depending on their z-positions within the channel. Furthermore, since the thickness of the channel (1.2 µm) is larger than half the wavelength of the excitation light in the host crystal ($\sim$ 250 nm), the channel contains several periods of this standing wave, meaning that molecules can, in principle, be at any position along the standing wave. In practice, due to the strongly focused nature of the excitation beam, we only need to consider pDCB-glass interfaces, as glass-vacuum interfaces are at least 150 µm away, much further than the Rayleigh range of the beam. Assuming the pDCB refractive index of 1.6, we obtain about 5 % amplitude reflection, which results in ±5 % amplitude modulation or, correspondingly, ±10 % power modulation. Since this modulation is position-dependent, we expect its effect to be different for different molecules, leading to overall spread but no systematic deviation.

- Refractive index uncertainty. Similarly to the detection-based method, one needs to know the refractive index of the host material. Given about ±5 % distribution for different crystal axes, we would expect this to produce ±10 % variation in the extracted quantum efficiency. Similarly to the other method, this will vary from molecule to molecule due to the difference in the local crystal orientation.

- Polarization-dependent losses in the illumination part of the setup. These can lead to variations in the incident power for differently oriented molecules, resulting in a miscalculation of the saturation power. We have estimated these power variations to be within ±10 % between different incident polarizations.

Finally, there are some common error sources, which contribute to both methods:

- Uncertainty in the branching ratio α. There are no clear studies of this value for DBT:pDCB. Since it is generally expected to depend on the host matrix, one can not readily use values obtained for other host crystals such as anthracene or naphthalene. Furthermore, one can not exclude intra-molecule variations, since this branching ratio can depend on the local phononic environment. On the other hand, this value contributes differently to the two methods: the

fluorescence-based emission method yields a signal proportional to $(1-\alpha)$, while the excitation method, which measures the strength of the ZPL, produces a signal proportional to α. Therefore, adjusting α to make both methods agree can minimize the effect of this uncertainty.

- Local photonic environment such as cracks in the crystal. They can lead to large deviations both in the excitation efficiency distribution and in the emission collection efficiency. We have taken two precautions to mitigate this effect. First, using an incoherent wide-field illumination and a camera we inspected areas around each molecule to make sure that there are no visible defects within several microns from the molecules. Second, we have confirmed that the illumination profile measured with our laser scanning method is well-approximated with an Airy disk.

Overall, the variations in the dipole emission collection efficiency and the residual uncertainty of about 10 % in the wavelength-dependent losses (based on the appearance of Fig. S3) yield the individual uncertainty of about 15 % for the first, emission-based, method. For the excitation-based method, the effects of interference, refractive index uncertainty, and polarization dependent losses are all estimated to be at around 10 %, leading to a total uncertainty of around 18 % for individual measurements. As we adjust the branching ratio to achieve best agreement between the two methods, the effect of the branching ratio is minimized, and the local crystal defects should not produce a large effect due to their rarity and additional inspection. Therefore, the overall uncertainty is predicted to range from 15 % to 20 %, which is consistent with the observed spread in Fig. 3(b).

[1] Glauber, R. J.; Lewenstein, M. Quantum Optics of Dielectric Media. *Phys. Rev. A* **1991**, *43*, 467–491.

[2] Rikken, G. L. J. A.; Kessener, Y. A. R. R. Local Field Effects and Electric and Magnetic Dipole Transitions in Dielectrics. *Phys. Rev. Lett.* **1995**, *74*, 880–883.

[3] Toptygin, D. Effects of the Solvent Refractive Index and Its Dispersion on the Radiative Decay Rate and Extinction Coefficient of a Fluorescent Solute. *J. Fluoresc.* **2003**, *13*, 201–219.

[4] Novotny, L.; Hecht, B. *Principles of Nano-Optics*; Cambridge University Press: Cambridge, U.K., 2006.

[5] Aubret, A.; Orrit, M.; Kulzer, F. Understanding Local-Field Correction Factors in the Framework of the Onsager- Böttcher Model. *ChemPhysChem* **2019**, *20*, 345–355.

[6] Zhang, P.; Ren, P. L.; Chen, X. W. On the Emission Pattern of Nanoscopic Emitters in Planar Anisotropic Matrix and Nanoantenna Structures. *Nanoscale* **2019**, *11*, 11195–11201.

[7] Wrigge, G.; Gerhardt, I.; Hwang, J.; Zumofen, G.; Sandoghdar, V. Efficient Coupling of Photons to a Single Molecule and the Observation of Its Resonance Fluorescence. *Nat. Phys.* **2008**, *4*, 60–66.

[8] Nicolet, A. A. L.; Hofmann, C.; Kol'chenko, M. A.; Kozankiewicz, B.; Orrit, M. Single Dibenzoterrylene Molecules in an Anthracene Crystal: Spectroscopy and Photophysics. *ChemPhysChem* **2007**, *8*, 1215–1220.
